# Spatial Patterns and Diversity of the Genus *Agave* in the Southern Iberian Peninsula: The Role of Anthropogenic Drivers in the Expansion of *Agave americana*

**DOI:** 10.3390/plants15020327

**Published:** 2026-01-21

**Authors:** Francisco Guerrero, Víctor Cid-Gaitán, Javier Jurado-Pardeiro, Fernando Ortega, Juan Diego Gilbert

**Affiliations:** 1 Departamento de Biología Animal, Biología Vegetal y Ecología, Universidad de Jaén, Campus de Las Lagunillas, s/n., 23071 Jaén, Spain; jpardeir@ujaen.es (J.J.-P.); fernandoortegagonzalez1972@gmail.com (F.O.); dgilbert@ujaen.es (J.D.G.); 2 Centro de Estudios Avanzados en Ciencias de la Tierra, Energía y Medio Ambiente, Universidad de Jaén, Campus de Las Lagunillas, s/n., 23071 Jaén, Spain; 3 Sociedad Ibérica para el Estudio y Conservación de los Ecosistemas (SIECE), Polígono Industrial Los Jarales, C/ Mina Alcolea, s/n., 23700 Jaén, Spain; vcgaitan@gmail.com

**Keywords:** *Agave americana*, cultural landscape, diversity, Mediterranean, species distribution modeling

## Abstract

The genus *Agave* L. is a key component of Mediterranean alien flora, yet its inland distribution in the Iberian Peninsula remains poorly understood. This research integrates exhaustive field surveys with Species Distribution Models (SDMs) to characterize the genus diversity and, specifically, the spatial patterns and environmental niche of *Agave americana* in the southern Iberian Peninsula (Andalusia). Our results reveal a diversity of 23 taxa, yet crucially, the widespread occurrence of *A. americana* demonstrated that its actual inland distribution is significantly more extensive than previously recorded. Spatial Point Pattern Analysis (SPPA) revealed a strong aggregated distribution pattern (Clark & Evans R = 0.277; *p* < 0.001). The MaxEnt Spatial Distribution Model demonstrated robust predictive performance (Mean AUC = 0.770 ± 0.007; Mean TSS = 0.420 ± 0.009). The distribution was primarily driven by elevation range (26.9%) and land use (23.1%), with maximum suitability peaking in anthropized, low-to-intermediate elevation areas. Projections to the broader Andalusian region confirmed high suitability in the Guadalquivir valley and coastal zones, validated by low spatial uncertainty (SD < 0.05 in optimal areas). These findings provide new insights into the biogeography of *Agave* in the region, emphasizing the significance of anthropogenic drivers within a cultural landscape context.

## 1. Introduction

The plant genus *Agave* L. is native to the Americas, with a natural distribution spanning from the southern United States through Central America to Bolivia and extending into the Caribbean islands [1,2,3]. This group comprises approximately 200 taxa, although the precise number remains variable and is often debated among different authors [4,5]. Currently, *Agave* species are distributed across all five continents, establishing a presence in numerous regions outside of their native range. In Europe, they have been formally reported in seven countries: Cyprus, Spain, France, the Netherlands, Italy, Portugal, and the United Kingdom [5].

However, the expansion of the *Agave* genus is not merely a botanical occurrence but a relevant ecological phenomenon within the Mediterranean Basin, a region identified as a global hotspot for biological invasions [6]. In these ecosystems, the introduction of exotic succulent species presents a duality: their functional traits—specifically high drought tolerance and clonal propagation—facilitate naturalization. Yet, the literature suggests they can simultaneously create new habitats, acting as ‘nurse plants’ that modify microhabitats and potentially interacting with native biodiversity [7,8]. Understanding these dynamics is essential, as the transition from introduced crops to naturalized components alters landscape functionality.

Their presence in the Iberian Peninsula has been documented since the 16th century [9,10,11]. Initial establishment was driven by ornamental appeal and fiber production [4], followed by integration into rural landscapes for traditional agricultural roles such as delimiting paths, defining property boundaries, or providing fodder. Building upon this historical baseline, recent expansion has been primarily fueled by modern landscaping and gardening [12]. Consequently, the current distribution does not contradict historical utilization but rather reflects the cumulative impact of these anthropogenic activities, resulting in the scattered and partially naturalized populations observed today.

Despite this history, current distribution maps primarily depict *Agave* presence in coastal provinces and archipelagos, with notably few records in the Iberian interior [13]. This distribution, heavily skewed towards coastal regions, likely underrepresents the legacy of extensive historical utilization across the broader Peninsula, reflecting a sampling bias and a lack of systematic prospection in inland territories rather than a genuine absence of the species. Indeed, recent field evidence gathered in the interior suggests a more widespread presence than currently mapped [14,15]. This lack of accurate distributional data creates a significant management blind spot, hindering the assessment of both invasion risks and potential ecosystem services.

Given the prevalence of the Mediterranean climate and bibliographic evidence of successful *Agave* naturalization in similar global regions [5,16], our hypothesis is that the actual distribution in the Iberian Peninsula is substantially more extensive than that recorded in official reports [13]. Therefore, the main objective of this study is to assess *Agave* diversity and distribution in the southern Iberian interior. Specifically, this study’s aims are as follows: (i) conduct a systematic field survey of all *Agave* taxa in the province of Jaén to characterize local diversity; (ii) develop Species Distribution Models (SDM) specifically for *Agave americana* to identify the environmental and anthropogenic drivers of its distribution; (iii) project the potential distribution of *A. americana* across the entire Andalusia region to provide a robust predictive baseline; (iv) provide key insights for management, moving beyond simple occurrence records towards a comprehensive ecological understanding of potential expansion.

## 2. Results

### 2.1. Spatial Distribution and Structure

**Figure 1:**
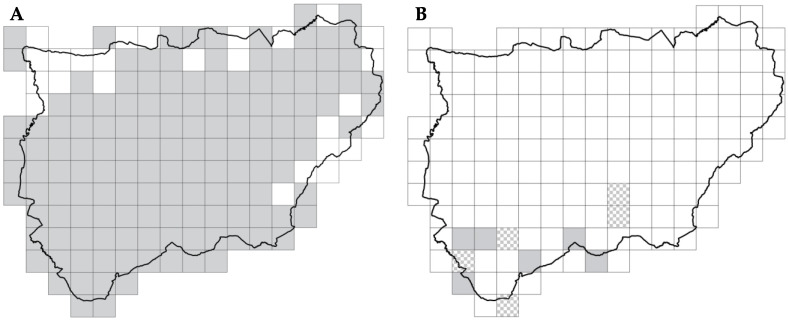
Positive presence records (gray squares) for *Agave* taxa (**A**) and literature data (**B**). The literature data include positive presence records from Sanz-Elorza and collaborators [10] (gray squares) as well as potential presence records from Gassó and collaborators [17] (gray and white squares).

**Figure 2:**
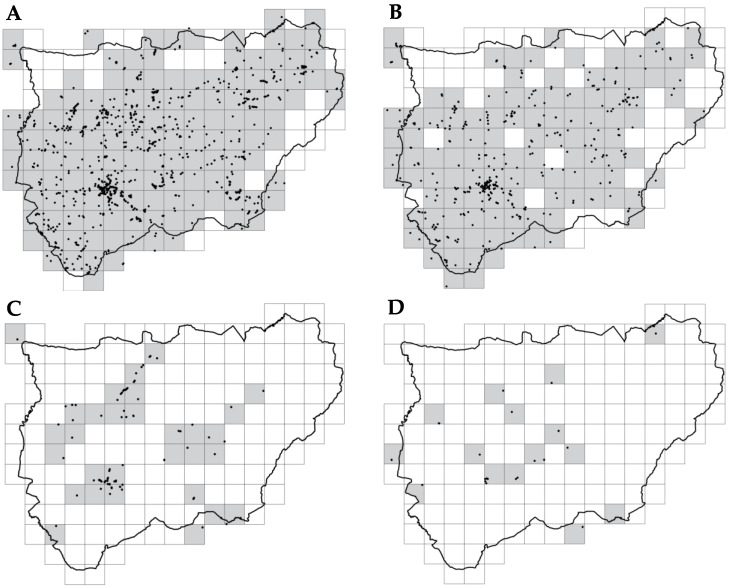
Points and grids showing the presence of *Agave* taxa: *Agave americana* var. *americana* (**A**); *Agave americana* var. *marginata* (**B**); *Agave ingens* var. *picta* (**C**); other *Agave* taxa (**D**). Black dots indicate specific occurrence records, and gray squares represent positive presence in 10 × 10 km grids.

The systematic field survey revealed a remarkably high diversity regarding the genus *Agave* in the study area, identifying a total of 23 taxa (Table 1). This taxonomic richness significantly exceeds previous records for inland territories. *Agave* specimens were recorded in 143 of the 169 studied 10 × 10 km grids (Figure 1A), with *Agave americana* var. *americana* being the most widespread taxon in the territory (Figure 2A), present in 138 grids. It was followed by *Agave americana* var. *marginata*, found in 114 of these grids (Figure 2B). Next, *Agave ingens* var. *picta* was present in 30 grids (Figure 2C), with other *Agave* taxa observed in 15 grids (Figure 2D). From this list, only *A. americana* (including its varieties *americana* and *marginata*) was widely naturalized within the study area. Consequently, subsequent modeling analyses focused exclusively on this taxon, as the remaining species presented insufficient occurrence records to meet the sample size thresholds required for robust SDM predictions. The distribution of these other taxa remains much more restricted and primarily associated with ornamental use.

**Figure 3:**
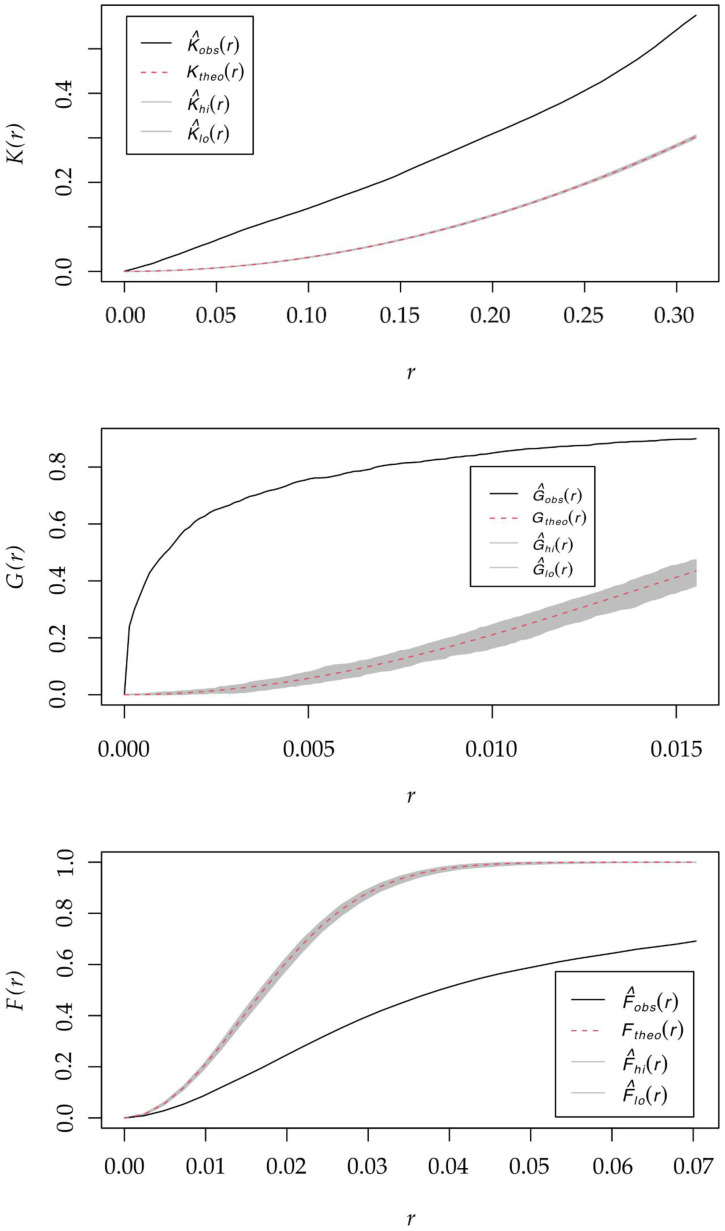
Results of Ripley’s functions: the K-function (point density), G-function (nearest neighbor distance), and F-function (empty space analysis) for *A. americana* in the study area. The solid black line represents the observed spatial pattern. The gray-shaded envelopes represent the 99% confidence interval under the null hypothesis of complete spatial randomness (CSR).

Regarding the spatial structure, Figure 3 shows the results of Ripley’s functions: the K-function (point density), the G-function (nearest neighbor distance), and the F-function (empty space analysis), for records of the *A. americana* taxon detected in the study area. The spatial distribution pattern significantly deviated from the null hypothesis of complete spatial randomness (CSR). The global Clark & Evans nearest neighbor index (R) consistently indicated strong aggregation (clustering; R = 0.277; *p* < 0.001). This R-value falls well below the typical range for random distributions, denoting a high intensity of clustering [18]. This pattern is consistent with limited dispersal mechanisms typical of clonal species or localized anthropogenic introduction, although we acknowledge that environmental heterogeneity in suitable microsites may also contribute to this arrangement.

Complementing this, the Quadrat test independently confirmed this highly aggregated structure with a substantial deviation from randomness (χ^2^ = 5221.5; *p* < 0.001). This consistent finding was further supported by Ripley’s K and G functions, where the observed curves lay significantly above the upper simulation envelopes (*p* < 0.01). The F function curve simultaneously fell below the lower envelope (*p* < 0.01) across nearly all tested distance scales. Collectively, these results confirm a robust, multi-scale clustered distribution characterized by small inter-individual distances and large empty spaces between clusters.

### 2.2. Model Performance and Habitat Suitability

The SDM for the *Agave americana* taxon was constructed using an ensemble approach involving 10 MaxEnt replicates, trained on 1340 thinned presence records using optimized settings (feature class: lqh; regularization multiplier (λ): 0.1). The ensemble exhibited acceptable performance metrics, demonstrating stability across all iterations. Specifically, the model achieved a mean area under the curve (AUC) of 0.770 ± 0.007 (mean ± standard deviation), indicating a fair discriminatory ability between suitable and unsuitable habitats. The classification performance, evaluated using the Kappa statistic (Cohen’s Kappa), yielded a mean value of 0.423 ± 0.015. Additionally, to ensure a robust evaluation of classification accuracy independent of prevalence, the True Skill Statistic (TSS) was calculated, yielding a mean value of 0.420 ± 0.009. We interpreted these metrics as indicative of a moderate predictive performance, sufficient for identifying regional suitability patterns but acknowledging the inherent complexity of modeling a widespread generalist species. This binary classification utilized an optimal threshold of 0.413 ± 0.018, determined by maximizing the Kappa value. This thresholding method was selected to optimize the trade-off between sensitivity and specificity, minimizing the risk of overestimating distribution in this invasion risk assessment.

The relative importance of the nine final environmental predictors, which were pre-processed and standardized to a consistent 1 km spatial resolution (see Table S1 for the definitions), was assessed using the mean percentage contribution across the 10 ensemble replicates (Table 2). The model’s predictive performance was predominantly driven by elevation range (26.89%) and land use (23.1%), which collectively accounted for half of the explained variation. Secondary drivers included terrestrial physiography (11.83%), lithological unit (10.38%), and biogeographical unit (9.48%). The remaining four variables, including the two standard bioclimatic predictors (temperature and precipitation), exhibited marginal contributions (<18%). While this suggests that distribution is primarily constrained by topographical and anthropogenic factors, we acknowledge that the 1 km spatial resolution and the dominance of categorical land-use predictors may mask fine-scale microclimatic influences.

The interpretation of variable response curves provides critical insight into the ecological niche of *A. americana*. Elevation range was the most influential predictor. Habitat suitability showed a unimodal response to altitude, peaking at 400–499 m a.s.l. (probability ∼0.294) and remaining moderate up to 900 m before dropping significantly above 1300 m. Crucially, the maximum probability for elevation alone (0.294) did not exceed the classification threshold (>0.413). This indicates that, despite being the primary predictor, elevation is insufficient to predict presence in isolation; high suitability requires synergy between optimal altitudes and favorable conditions in other variables (e.g., land use).

**Figure 4:**
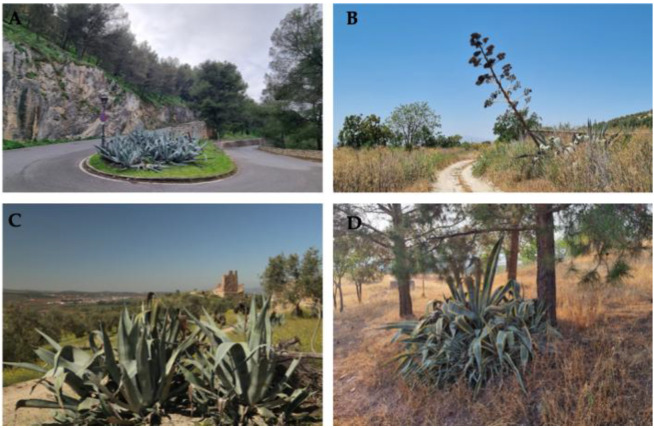
Representative images of *A. americana* in the study area: as an ornamental element (**A**), along a roadside (**B**), in an agricultural setting (**C**), and in a forested environment (**D**).

*A. americana* shows a notable capacity for persistence across human-modified environments (Figure 4). The highest suitability was recorded in sports and recreational facilities (suitability of ∼0.4943), likely driven by deliberate ornamental introduction (xeriscaping). Other categories providing relatively high habitat suitability included road and rail networks and associated land (predicted suitability ∼ 0.4389), folded mountain reliefs in conglomerate and granular rocks (∼0.4320), agricultural lands with significant natural vegetation (∼0.4205), and continuous urban fabric yielded (predicted suitability ∼ 0.4137).

**Figure 5:**
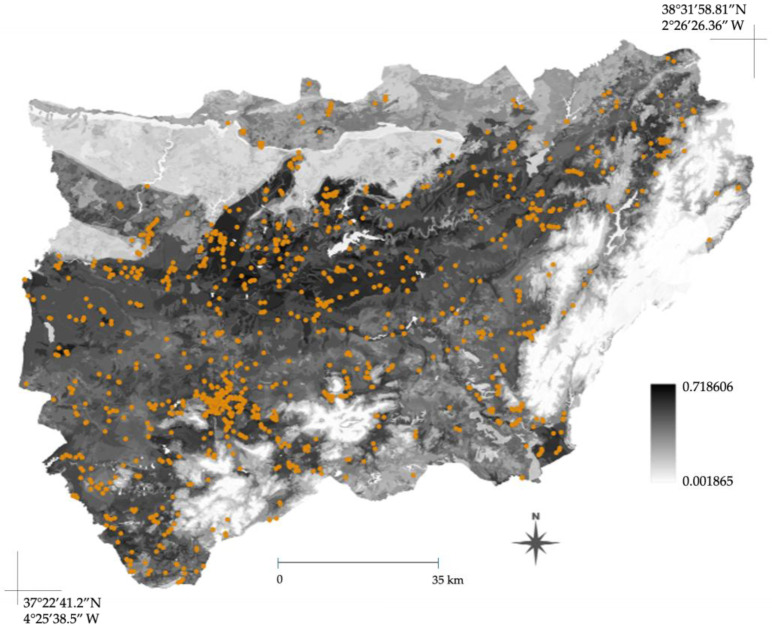
Continuous-occurrence probability map generated by the ensemble species distribution model (optimized MaxEnt) for *A. americana* in Jaén province. Grayscale values indicate environmental suitability, ranging from light (low suitability) to dark tones (optimal habitat suitability). Overlaid orange points denote the observed species occurrence records used for model training, demonstrating strong spatial agreement with the regions of highest predicted habitat suitability.

The continuous spatial projection of the MaxEnt ensemble model (Figure 5) visualizes predicted environmental suitability, confirming the influence of the elevation and land use. Figure 5 shows a highly heterogeneous pattern, with the highest environmental suitability concentrated predominantly in the Guadalquivir valley and central/south-western quadrants. These zones correspond to low-to-intermediate altitudinal ranges and human-modified areas. Conversely, the model predicted the lowest suitability (light to white tones) across the eastern portion and north-eastern margins. This clear boundary correlates with high-altitude areas, confirming the species’ low tolerance for high-elevation conditions, and with areas of less anthropic influence.

**Figure 6:**
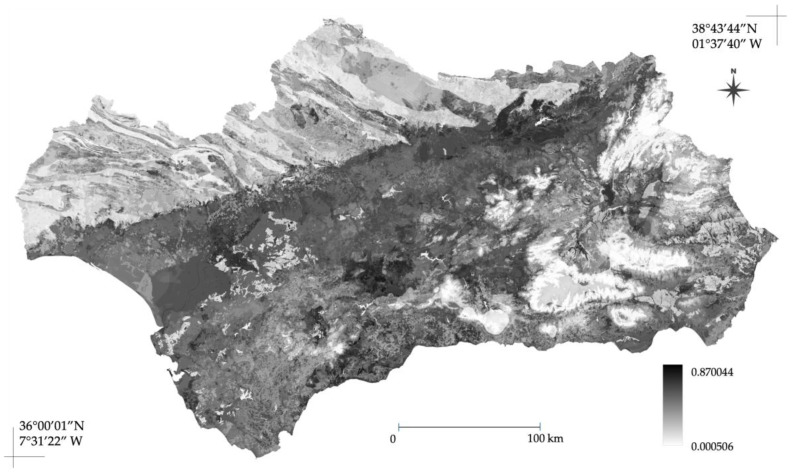
Continuous spatial projection of the *A. americana* species distribution model (SDM) across the Andalusia region. The map displays the environmental suitability (predicted probability of presence) generated by the Maxnet ensemble model, with values ranging from 0 (unsuitable) to 1 (optimal suitability).

Finally, the projection across Andalusia (Figure 6) revealed similar patterns. The highest suitability values (>0.8) are concentrated across the major low-altitude areas of the region, notably the Guadalquivir valley and coastal plains of Cádiz and Huelva, reflecting the species’ association with intermediate-to-low elevation and agricultural matrices. Conversely, low suitability strictly delimits high-elevation mountain systems (Sierra Morena and the Baetic ranges), where the species’ presence is severely restricted.

### 2.3. Evaluation of Model Robustness and Spatial Uncertainty

**Figure 7:**
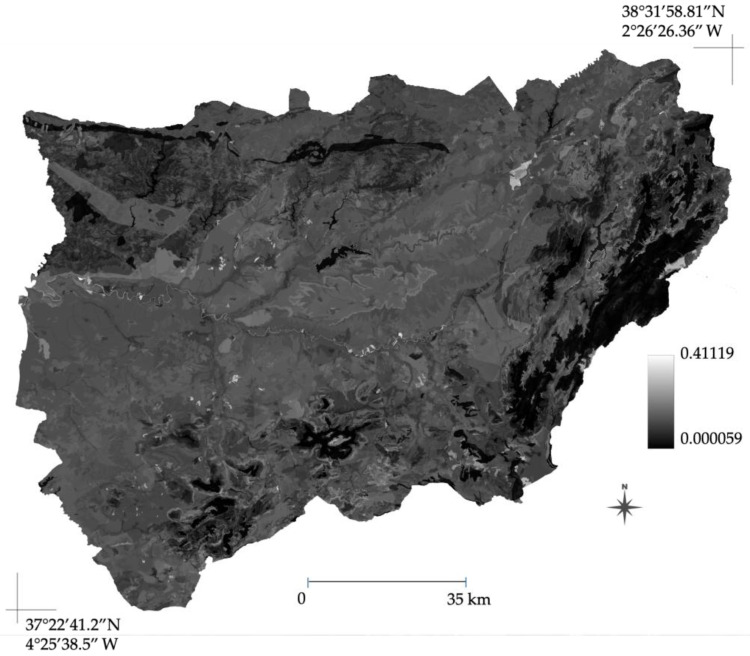
Spatial uncertainty map for the *A. americana* distribution model within the calibration area (Jaén province). The map displays the pixel-wise standard deviation (SD) calculated across the 10 independent MaxEnt ensemble replicates. Lower SD values (darker tones) indicate areas of high model consensus and internal stability, confirming that model predictions were consistent across iterations.

The assessment of spatial uncertainty, calculated as the pixel-wise standard deviation (SD) across the 10 ensemble replicates, confirmed the stability of the model predictions. In the calibration area (Jaén; Figure 7), regions identified as highly suitable for *A. americana* consistently exhibited very low uncertainty values (mean SD < 0.05), indicating a high degree of consensus among the model iterations.

**Figure 8:**
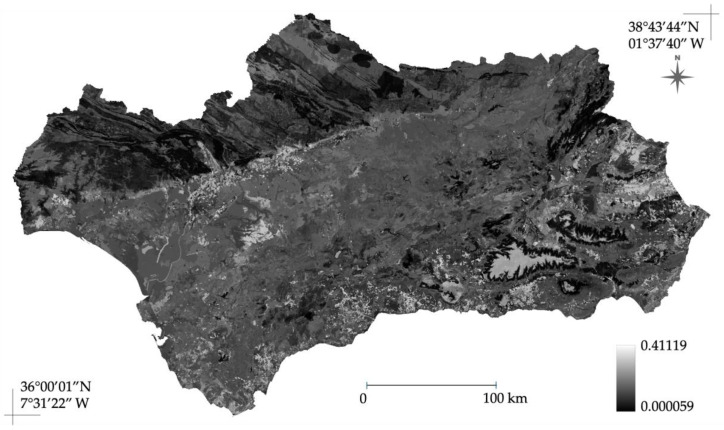
Spatial uncertainty map for the *A. americana* model projected across the broader Andalusian region. Uncertainty is quantified as the pixel-wise standard deviation (SD) across the 10 ensemble replicates extrapolated to the regional extent. Low SD values indicate high consensus in predicting suitable habitats (e.g., in the Guadalquivir valley and coastal zones), while higher values reflect predictive divergence in transitional or environmental extrapolation zones.

When projected to the broader Andalusian region (Figure 8), the model maintained its robustness, with maximum suitability areas (specifically the Guadalquivir valley and coastal plains) coinciding with zones of minimal divergence. Notably, while the spatial patterns of uncertainty within the Jaén province were consistent between the calibration and projection maps, the absolute pixel values exhibited slight variations. This difference is expected and attributable to the transition from the native calibration grid to the broader regional projection grid, where the model algorithms interact with a standardized environmental stack of different extent. However, it is important to interpret these results with caution, as low standard deviation reflects the high internal consistency of the ensemble replicates rather than an independent validation of the model’s transferability to novel environments outside the calibration range.

## 3. Discussion

### 3.1. Ecological Results: Diversity and Comparison with Other Regions

The research data identified a high number of species in the study area, with 23 total taxa located, and *A. americana* exhibiting the greatest territorial distribution and highest degree of naturalization. The total number of species is substantial given the limited size of the study area (13,489 km^2^). This density contrasts sharply with reports from other regions where *Agave* has been introduced, such as Eastern Cape province of South Africa (170,000 km^2^), which records only five naturalized *Agave* species [19]; the Algarve region of Portugal, another Mediterranean area, which reports only four naturalized *Agave* species [16]; and even the Canary Islands, which support 15 *Agave* species [20].

Furthermore, these values are still remarkably high even when compared to the native range of the genus in Mexico, where species richness in individual states ranges between 20 and 43 species [21]. However, this comparison requires careful interpretation because while diversity in the native range is the result of evolutionary speciation, the concentration found in our study area represents an anthropogenic accumulation. We attribute this exceptional density to the convergence of two key factors: firstly, the intense ornamental introduction pressure driven by esthetic preferences that actively recruit a wide variety of taxa into the landscape; secondly, the local Mediterranean climate, which provides a suitable niche highly conducive to the long-term survival and vegetative persistence of these species.

### 3.2. Anthropogenic Drivers of Distribution

The wide and aggregated distribution of the *Agave* genus is spatially structured by a temporal shift in anthropogenic drivers. Initially, cultivation for fiber—a practice inherited from its native range [22]—established a spatial configuration in the Iberian Peninsula restricted to agricultural terraces and peri-urban plots near settlements. However, our results indicate that recent ornamental demands have reconfigured this pattern, positioning land use as a major determinant of the species’ distribution [23]. High suitability was recorded in the following: (i) sport and recreational facilities; (ii) road and rail networks and associated lands; (iii) agricultural landscapes; (iv) continuous urban fabric. Notably, the peak suitability in sport and recreational facilities indicates a management-driven distribution rather than a preference for specific substrates. These facilities often incorporate low-maintenance ornamental vegetation (xeriscaping) to reduce irrigation costs in the Mediterranean summer; consequently, *A. americana*, with its drought tolerance, acts as an ideal candidate [16]. Thus, these recreational zones function as anthropogenic refuges and active dispersal hubs, explaining the high predicted occurrence despite the artificial nature of the substrate. In contrast, presence in other landscapes remains associated with past human activities. The close relationship with communication routes (road and rail networks) marks the historical onset of its territorial redistribution, mirroring spatial patterns established by historical paths like Mexico’s Camino Real [24]. From these primary lines, dispersal into the broader anthropic landscape was gradual, driven by new activities and the expansion of resource availability [25].

Our results identify elevation range as the dominant environmental predictor, with a mean contribution of 26.89% and a distinct suitability peak at intermediate altitudes (400–499 m a.s.l.) in the Jaén province (Table 2). However, this factor alone is insufficient to generate a robust positive prediction, necessitating a synergistic effect with other environmental variables, and emphasizing the multicausal nature of the distribution. We hypothesize that elevational range likely acts as a surrogate for anthropogenic presence rather than indicating a direct physiological barrier. Although the model highlights altitude as a limiting factor, the underlying mechanism appears to be biotic: in the study area, traditional agricultural activity and settlements—the obligatory vectors for the historical *Agave* dispersal—are concentrated within this elevational belt. Consequently, the sharp decline in suitability at higher elevations is consistent with a lack of propagule pressure (human-mediated dispersal) rather than thermal intolerance, a pattern that aligns with the aggregated spatial structures observed (Figure 3). While altitude is strongly correlated with decreasing temperature, potentially limiting expansion, physiological evidence refutes this as the primary constraint. *A. americana* exhibits significant cold acclimation, tolerating temperatures down to −10 °C [26], and its established presence in colder regions of Spain [15] confirms that thermal conditions in the study area are not exclusionary. Similarly, its broad tolerance to precipitation variability and drought [21,27] minimizes the restrictive role of water availability. Instead, the distribution appears driven by land-use intensity. This aligns with broader Mediterranean patterns where artificial dry habitats host significantly higher alien species richness (approx. 550 species) compared to natural habitats (approx. 150 species) [28]. Crucially, high-altitude zones in the study area coincide with Natural Parks; these protected areas, characterized by minimal anthropogenic disturbance, inherently minimizes the presence of these non-native species.

Finally, although specific quantification was outside the scope of this study, we hypothesize that herbivory functions as an additional demographic filter restricting presence in elevated natural areas, a mechanism framed within the biotic resistance hypothesis [29]. Unlike the agricultural lowlands, these mountain territories sustain high densities of wild ungulates (e.g., *Capra pyrenaica*) and extensive livestock. Grazing pressure affects the species across its life cycle; while adult *Agave* specimens are mechanically protected by spines, young clonal shoots and bulbils—crucial for colonization—are soft, highly palatable, and vulnerable to browsing [30]. This predation likely acts as a demographic bottleneck, successful establishment in open, grazed ecosystems.

### 3.3. Cultural Context and Social Perception

The widespread presence of the genus *Agave* across the study area has had a significant impact on local society, which largely perceives these species—specifically the *A. americana* taxon—as a natural component of the environment [31]. Currently, *Agave* species are present in the majority of landscapes within the study area [31,32]. In this context, it is crucial to recognize that the territories of Jaén and Andalusia are effectively cultural landscapes rather than pristine natural environments [33]. This long-standing interaction between humans and nature, coupled with the prevailing environmental conditions of the Mediterranean context, has positioned this region as a global biodiversity hotspot [34]. Naturalized alien species contribute to this richness, although they are frequently targeted for eradication by management agencies. We argue that such policies, while ecologically motivated, may inadvertently neglect the historical integration of these species and the potential ecosystem services they provide. Historically, this cultural integration was driven by fiber extraction—an activity now preserved by few artisans—and boundary demarcation. More recently, this dimension has evolved to include ornamental use and even esthetic roles in the historic cinematographic settings of Almería province.

### 3.4. Ecological Implications and Ecosystem Services

Beyond the anthropogenic drivers and cultural context, there is increasing evidence that *Agave* species provide significant ecosystem services in these introduced ranges. While studies in the Canary Islands (Spain) have highlighted the importance of *Agave* nectar and pollen for endemic avifauna [7], our observations in the study area identify two distinct functional roles. First, the plant serves as a trophic resource for both wild and domestic herbivores during periods of feed scarcity. Second, the structural complexity of mature specimens facilitates the formation of subterranean shelter systems beneath the canopy. These micro-habitats act as refuges for local fauna, where the spiny rosette serves as a deterrent against potential predators [35]. Consequently, rather than viewing these populations solely as biological invasions, we propose considering them as functional elements of the Andalusian cultural landscape, a perspective that could inform more balanced conservation polices.

### 3.5. Regional Projection and Conservation Challenges

The environmental predictors identified are clearly evident in the distribution maps for both the study area (Jaén province) (Figure 5) and the projection across the broader Andalusia region (Figure 6). This projection demonstrates that areas with higher population density and human intervention—primarily the Guadalquivir River valley and coastal zones—exhibit greater suitability. Suitability subsequently decreases toward the surrounding mountainous territories: Sierra Morena to the north and the Baetic Ranges to the south. Furthermore, the spatial uncertainty analysis (Figure 7 and Figure 8) revealed a low standard deviation across most of the study area, indicating a high degree of consensus among the 10 ensemble replicates. This predictive stability reinforces the reliability of the identified suitable areas [36], confirming that the projected distribution is robust and not an artifact of the random selection of background points. It is noteworthy that, despite the model being calibrated within a territory where the minimum altitude exceeds 150 m a.s.l., it demonstrates robust performance when extrapolated to the entire region. Crucially, the model reiterates high environmental suitability in coastal areas, a finding consistent with both the published literature [10] and prior distribution models [17].

However, recent global changes within the context of the Anthropocene [37] are driving biodiversity loss [38] and landscape homogenization, often characterized by intensive resource exploitation that can lead to both cultural and ecological impoverishment [39]. Compounding these anthropogenic pressures is the rapid expansion of the agave snout weevil (*Scyphophorus acupunctatus* Gyllenhal), across Andalusia [40]. Given these threats, the future of the specific ecological and cultural landscape provided by agaves is uncertain. We suggest that a sustainable management approach should integrate both natural and cultural landscape values. This holistic perspective would weigh ecological risk against cultural heritage [41,42]. In practice, this implies moving beyond indiscriminate eradication toward specific management guidelines, such as the active monitoring of the weevil’s expansion and the selective conservation of historic populations in cultural settings.

Finally, beyond the specific case of *A. americana*, the methodological framework employed here—integrating anthropogenic drivers with environmental predictors—offers a robust and replicable approach to study the distribution dynamics of other naturalized taxa in complex cultural landscapes.

## 4. Materials and Methods

### 4.1. Study Area and Data Collection

**Figure 9:**
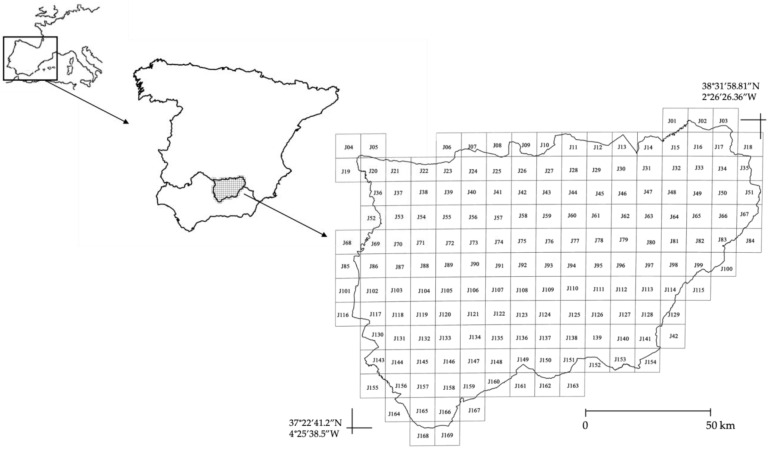
Study area in the southern Iberian Peninsula (Andalusia and Jaén province), showing the systematic division into 169 grid cells used for the comprehensive field survey in Jaén. The numbers overlaid on the Jaén province map correspond to the unique identifier assigned to each grid cell.

The study focuses on the province of Jaén, located in the southeastern Iberian Peninsula (Andalusia, Spain; Figure 9). The territory spans a latitudinal range of 37°22′41.2″–38°31′58.81″ N and a longitudinal range of 2°26′26.36″–4°25′38.5″ W, covering an elevational gradient from approximately 200 to 2150 m a.s.l. Despite the literature suggesting a limited presence of the genus *Agave* in this region (Figure 1), we conducted an extensive sampling effort across the entire territory. Fieldwork conducted from April 2021 to August 2025 allowed us to locate isolated individuals and populations and determine species diversity.

The territory was divided into 169 grids using the 10 × 10 km Universal Transverse Mercator (UTM) system (Figure 9), which facilitated the assessment of the presence or absence of different *Agave* species. While we acknowledge that maintaining a perfectly constant sampling intensity over a four-year period is logistically challenging, this grid-based strategy was designed to approximate a homogeneous sampling effort across the study region [43,44,45]. Unlike opportunistic datasets, this systematic design minimizes environmental bias, ensuring that inferred species–environment associations reflect true ecological patterns rather than artifacts of unequal sampling intensity.

For distribution modeling, *Agave* occurrences were geolocated using a Global Positioning System (GPS) during field surveys and identified as specific varieties of *A. americana*. This continuous and spatially extensive sampling effort allowed for a detailed characterization of the entire study area, minimizing the potential biases often associated with non-systematic data collection.

### 4.2. Spatial Pattern Analysis

To evaluate the spatial distribution of *A. americana* taxon, a spatial point pattern analysis (SPPA) was performed using the spatstat package (v. 3.3-3) in R (version 4.5.1). Occurrence records were converted into a point pattern object defined by their longitude and latitude coordinates. The observation window (owin) was set as the bounding box of the point occurrences, incorporating a minimal buffer to mitigate edge effects. The analysis assessed whether observed distributions deviated significantly from the null hypothesis of CSR, typically modeled by a homogeneous Poisson process. Three complementary methods were employed: (i) Ripley’s K-Function K(r), used to assess the scale-dependent spatial structure by measuring the expected number of neighboring points within a distance r of an arbitrary point. The observed K(r) curve was compared against envelopes generated from 99 Monte Carlo simulations under CSR. Values above the upper envelope indicate clustering, while those below imply regularity. (ii) Clark & Evans nearest neighbor index (R), calculated to quantify global departure from CSR. R represents the ratio of the mean observed nearest neighbor distance to the expected mean under CSR. Values of R < 1 indicate clustering, whereas R > 1 indicates regularity. Significance was tested using a two-sided Z-test. (iii) Quadrat test, a chi-square (χ^2^) test conducted to evaluate CSR globally. The study area was divided into a 5 × 5 grid, and the observed point counts per quadrat were compared to the expected counts. A *p*-value < 0.05 was used to reject the hypothesis of randomness.

### 4.3. Species Distribution Models

SDMs, fundamental tools in biogeography and ecology [46,47,48,49], operate by correlating species occurrence with environmental predictors [49] to predict species presence or relative habitat suitability across unsampled geographic areas [50]. Our analysis followed the standard four-phase procedure of SDM construction: (i) data gathering based on precise GPS records; (ii) model building; (iii) model evaluation; (iv) spatial projection [46,47,51,52].

Model construction relied on high-accuracy GPS occurrences to minimize positional noise. To mitigate the bias associated with opportunistic sampling [53,54,55,56], a systematic sampling approach was adopted. As noted, this grid-based effort assumes a relatively homogeneous sampling effort across the study region [43,44,45]. Species presence was recorded in each cell occupied by *A. americana*, with the resulting presence data serving as the dependent variable for the model. The final dataset used for model calibration comprised 1770 unique presence records. Given the reliance on presence-only data, a parallel set of contrast data was required for model training. These contrast data points, often termed pseudo-absences (PAs) or background points [57], were generated using the random sampling (RS) method, which is recognized as the simplest and most frequently utilized approach for PA generation [58]. Specifically, a total of 10,000 background points were randomly generated across the study area, excluding cells containing presence records to avoid false negatives.

Environmental predictors were selected to characterize the climatic and topographic heterogeneity of Andalusia. Nine Geographic Information System (GIS) layers (detailed in Table S1) were sourced from the Institute of Statistics and Cartography of Andalusia (https://www.juntadeandalucia.es/institutodeestadisticaycartografia/dega/datos-espaciales-de-referencia-de-andalucia-dera/ accessed 26 September 2025). These included two continuous variables (precipitation and temperature) and seven categorical variables (territorial domain, terrestrial physiographic unit, altitude range, landscape, biogeographic unit, lithological unit and land use). All layers were pre-processed and resampled to a consistent spatial resolution of 1 km. To avoid multicollinearity, variables with a variance inflation factor (VIF) > 5 were removed using the fuzzySim::multicol function.

Models were built using the maximum entropy (MaxEnt—[59]) algorithm, selected for its high performance with presence-only data [60] and robust regularization capabilities [61]. The algorithm was implemented via the maxnet package in the R statistical environment. The optimization process involved a grid search across two main hyperparameters: the feature classes (FCs) and the regularization multiplier (λ). The search space for FCs included combinations of linear (L), quadratic (Q), hinge (H), product (P), and threshold (T) features. The λ parameter was tested with values 0.1, 0.5, 1.0, 2.0, and 4.0. Each hyperparameter combination was evaluated using 5-fold cross-validation, and the optimal set (FC and λ) was selected as the one that maximized the mean AUC across the five test folds.

Model performance was assessed using three standard metrics (mean ± SD across 10 iterations). First, the AUC quantified the model’s discrimination capacity, interpreted following established criteria by Swets [62]. Second, the Kappa statistic was used as the primary measure of model agreement. The optimal Kappa threshold was subsequently used to explicitly define the critical environmental niche boundaries for *A. americana*. This threshold served as the minimum acceptable probability for any environmental condition to be considered suitable. For categorical variables (e.g., land use), only the specific levels whose predicted logistic probability surpassed this threshold were deemed suitable. For continuous variables, the threshold was applied to the response curves to identify the precise optimal range of values where the predicted probability of occurrence was equal to or greater than the Kappa threshold. Third, the TSS was calculated as sensitivity + specificity −1 to provide a prevalence-independent measure of predictive accuracy [63]. Variable contribution was quantified using permutation importance and the Jackknife procedure [59].

To ensure model stability and robustness, the final MaxEnt prediction was generated as an ensemble model comprising 10 independent replicates, each trained on a new set of PAs. The nine pre-processed environmental layers for Andalusia, which had been standardized and aligned to a 1 km resolution, were combined into a final multi-layer raster stack. Model application was performed using the terra::predict() function within the R environment, applying the optimized Maxnet model to this predictor stack. The type=“logistic” argument was employed to obtain continuous predictions of habitat suitability. The final consensus prediction map represents the mean probability of these replicates. To rigorously assess robustness, we generated spatial uncertainty maps by calculating the pixel-wise standard deviation (SD) across the 10 independent ensemble replicates for both the calibration area (Jaén province) and the extrapolated projection across the entire Andalusian region. This metric identified areas of high model consensus (low SD values) versus areas of predictive instability, ensuring that the identified high-suitability zones were supported by consistent model agreement.

## 5. Conclusions

This study represents the first systematic assessment of the genus *Agave* in the southern Iberian interior, providing a new biogeographical baseline that significantly updates previous records. Our exhaustive field survey revealed an unexpectedly high diversity of 23 taxa, identifying the region as a significant reservoir of *Agave* diversity previously underestimated in official databases. However, beyond taxonomic richness, the most critical finding is the widespread naturalization of *A. americana* varieties, which have colonized the territory far more extensively than suggested by earlier theoretical models.

Our results challenge the prevailing view that restricted the invasion risk of *Agave* primarily to coastal areas due to climatic constraints. Instead, we demonstrate that anthropogenic drivers—specifically historical cultivation and land-use changes—act as primary forces facilitating the expansion of *A. americana* in inland ecosystems, enabling the species to persists in areas where purely climatic models might predict low suitability. This confirms that the “distributional gaps” observed in previous continental-scale maps were not due to an absence of the species, but to a lack of precise ground-truthing in the interior.

Consequently, these findings suggest the value of an integrated management perspective. Since the distribution of *Agave* is inextricably linked to human activity and cultural landscapes, conservation strategies might benefit from moving beyond a uniform “eradication” approach. We propose considering a dual strategy: active monitoring of pest expansion, such as *Scyphophorus acupunctatus*, and control in sensitive natural habitats, while simultaneously recognizing the cultural and ecological role of historical populations in rural agricultural systems. Ultimately, these findings provide the necessary framework to understand the colonization dynamics of succulent species in the Mediterranean, emphasizing that in highly anthropized landscapes, human history is as strong a driver of distribution as climate.

## Figures and Tables

**Table 1:** List of *Agave* taxa identified in the study area.

*Agave* Taxa	
*Agave americana* L. var. *americana*	*Agave ingens* Berger
*Agave americana* L. var. *marginata* Trel.	*Agave ingens* Berger var. *picta* (Salm-Dyck) Berger
*Agave americana* L. var. *medio-picta* Trel.	*Agave lechuguilla* Torr
*Agave angustifolia* Haw.	*Agave parrasana* Berger
*Agave angustifolia* Haw. var. *marginata* Trel.	*Agave parryi* Engelm var. *truncata* Gentry
*Agave asperrima* Jacobi	*Agave potatorum* Zucc
*Agave attenuata* Salm-Dyck	*Agave salmiana* Otto ex Dietr. var. *ferox* (Koch) Gentry
*Agave desmettiana* Jacobi	*Agave scabra* Salm-Dyck
*Agave desmettiana* Jacobi var. *marginata*	*Agave sisalana* Perrine
*Agave franzosini* (Sprenger) Sewell	*Agave titanota* Gentry
*Agave gentryi* Ullrich	*Agave weberi* Cels ex Poisson
*Agave gypsophilla* Gentry	

**Table 2:** Contribution (mean and standard deviation) of environmental variables to the *A. americana* MaxEnt ensemble model.

Variable	Mean Contribution	Standard Deviation
Elevation range	26.89	3.42
Land use	23.1	2.67
Terrestrial physiography	11.83	4.43
Lithological unit	10.38	3.23
Biogeographical unit	9.48	2.26
Landscape	5.9	3.13
Territorial domain	5.72	2.03
Temperature	5	1.83
Precipitation	1.7	1.83

## Data Availability

The original contributions presented in this study are included in the article/Supplementary Materials. Further inquiries can be directed to the corresponding author.

## Supplementary Materials

The following supporting information can be downloaded at https://www.mdpi.com/article/10.3390/plants15020327/s1. Table S1: Environmental predictor variables and their corresponding categorical classes used in SDMs for *A. americana* in Jaén and Andalusia.

## Abbreviations

The following abbreviations are used in this manuscript:

CSR	Complete Spatial Randomness
SDM	Species Distribution Model
AUC	Area Under the Curve
UTM	Universal Transverse Mercator
GPS	Global Positioning System
SPPA	Spatial Point Pattern Analysis
Kappa	Cohen’s Kappa
ENM	Ecological Niche Model
HSM	Habitat Suitability Model
PA	Pseudo-absence
RS	Random Sampling
GIS	Geographic Information System
VIF	Variance Inflation Factor
FC	Feature Class
ROC	Receiver Operating Characteristic
TSS	True Skill Statistic
